# PS-MPs Induced Inflammation and Phosphorylation of Inflammatory Signalling Pathways in Liver

**DOI:** 10.3390/toxics12120932

**Published:** 2024-12-22

**Authors:** Mengchao Ying, Naimin Shao, Cheng Dong, Yijie Sha, Chen Li, Xinyu Hong, Yu Ding, Jing Xu, Kelei Qian, Gonghua Tao, Ping Xiao

**Affiliations:** 1 Shanghai Municipal Center for Disease Control & Prevention, Shanghai 200336, China; 2 State Environmental Protection Key Laboratory of Environmental Health Impact Assessment of Emerging Contaminants, Shanghai 200233, China

**Keywords:** microplastics, interleukins, inflammatory cytokines, phosphorylation

## Abstract

As new pollutants, microplastics (MPs) have attracted much attention worldwide because they cause serious environmental pollution and pose potential health risks to humans. However, the toxic effects of MPs are still unclear. In this study, we analysed the inflammatory effects of 0.1 μm polystyrene microplastics (PS-MPs) on mouse and human liver cell lines. After 28 days of exposure to PS-MPs, the mice presented decreased liver index values and increased AST/ALT values. HL7702 and HepG2 were treated with PS-MPs for 24 h, and the cytotoxicity, the expression levels of inflammatory factors, and the phosphorylation of proteins in inflammation related pathways were confirmed. Compared with the control, the cell viability of these two cells significantly decreased after exposure to the PS-MPs at 1000 μm/cm^2^, and the BMD model also exhibited a similar dose. LDH leakage and AST also increased in a dose-dependent increase after PS-MPs exposure. The relative levels of chemokines such as GM-CSF, IL-6, IL-8, and IL-12p70 were significantly greater than those in the control. Furthermore, the PS-MPs can increase the expression levels of *TLR4*, *MyD88,* and *NF-κB* and activate the phosphorylation of NF-κB and STATs. Based on these results, exposure to PS-MPs can stimulate liver inflammation and activate the TLR4/MyD88/NF-κB and JAK-STAT pathways.

## 1. Introduction

Plastic, as a ubiquitous and indispensable part of daily life, is widely used everywhere. However, since Azzarello et al. reported that plastic waste may be misunderstood as prey and ingested by seabirds, much attention has been given to plastic litter [1]. In 2011, the European Union (EU) released several reports and documents to emphasize the issue of plastic waste, which included the increasing attention to plastic debris in the ocean, detection methods, and pollution control of plastic pollution [2]. Since then, people have focused on even smaller microplastics (MPs). Several methods and studies related to the identification and quantification of MPs pollution in the ocean have been reported. Usually, large plastic fragments can decompose into smaller fragments under a series of physical and chemical conditions. In 2004, Thompson et al. first reported and proposed the concept of MPs and isolated plastic debris with a size of approximately 20 μm in the ocean [3]. MPs are generally defined as plastic particles with a size of less than 5 mm. The main components of MPs include polyethylene (PE), polypropylene (PP), polystyrene (PS), polyvinyl chloride (PVC), polylactic acid (PLA), and polyethylene terephthalate (PET) [4]. In 2008, the United States convened the first meeting to assess the risk of interactions between MPs and animals. Many studies have suggested that MPs cause serious environmental pollution problems in water, soil, and air [5,6,7]. Although the majority of MPs studies have focused on the harmful effects of pollution on ocean and water quality, recent studies have investigated the effects of MPs in terrestrial ecosystems. For example, submicron- and micron-sized MPs can penetrate and enter crops such as wheat and lettuce [8]. Furthermore, MPs have also been detected in plastic products or foods that are generally considered safe, such as plastic feeding bottles, honey, and table salt for infants, and the amount of MPs contained in infant faeces is greater than that in adult faeces [7,9,10]. Several studies have also shown that MPs can be detected in blood and placental tissue [11,12]. Although these data indicate that our emphasis on MPs needs to be strengthened, the potential toxicity of MPs is still unclear. Therefore, it is necessary to study the potential hazards of MPs and their effects on human health.

Currently, a number of studies have shown that PS-MPs can cause damage to liver tissue and may induce the development of adverse outcomes such as hepatocellular carcinoma (HCC) and liver fibrosis. Zhao et al. reported that 0.5 μm PS-MPs induced AST, ALT, and TBIL in mouse serum after 28 days of oral exposure. The mRNA expression levels of IFN-γ, TNF-α, IL-1β, IL-6, and IL-33 are also increased, whereas those of IL-4, IL-5, IL-10, IL-18, and TGF-β1 are reduced after PS-MP exposure [13]. Other studies in liver organoids showed that AST and ALT activity and IL-6 expression were significantly upregulated after 0.1 μm PS-MPs treatment. PS-MPs can also induce ROS production and then cause oxidative stress and inflammatory responses [14]. Due to the presence of traces of polystyrene in the environment and human body and the ease of material synthesis and modification, PS-MPs have been widely used in MPs-related research as a standard substance. Although this study did not reveal significant novelty in the toxic effects of MPs, this study used HL7702 and HepG2 human liver cell lines exposed to 0.1 μm PS-MPs and performed confirmatory tests to confirm the cytotoxicity, the expression of proinflammatory proteins, and the phosphorylation levels of key factors in related signalling pathways.

## 2. Materials and Methods

### 2.1. Polystyrene Microspheres

The 0.1 µm unlabelled monodisperse PS-MPs microspheres were purchased from BaseLine Chromtech Research Centre (Tianjin, China), and the 0.1 µm green fluorescently labelled monodisperse PS-MPs microspheres were purchased from Beijing Zhongkeleiming Technology Co., Ltd. (Beijing, China).

### 2.2. Animal Experiments

All the protocols of the animal experiments were based on OECD Test No. 407: Repeated Dose 28-day Oral Toxicity Study in Rodents [15] and approved by the Animal Ethical and Welfare Committee of Shanghai Municipal Centre for Disease Control and Prevention (Approval No. IACUC-PZ-041-2024). Eight-to-ten-week-old C57BL/6J mice (half male and half female) were housed in groups of five per cage under a 12/12 h day/night cycle, and the environment was maintained at 40–70% relative humidity and 22 ± 3 °C. All the mice were divided into control and experimental groups (*n* = 10). A total of 0.5 mg of unlabelled PS-MPs was diluted with 100 μL of ddH_2_O and then administered to the experimental mice by gavage (100 μL/day). After the 28 d experiment, the mice were euthanized via isoflurane inhalation, and the body and liver weights were measured.

### 2.3. Serum Liver Function Indicators and Hepatic Pathology

The blood samples were collected into nonanticoagulant tubes and centrifuged to separate the serum. The aminotransferase (AST), alanine aminotransferase (ALT), alkaline phosphatase (ALP), total protein (TP), albumin, total bilirubin (TBIL), blood urea nitrogen (BUN), triglycerides (TG), cholesterol (CHOL), P^5+^, Ca^2+^, K^+^, Na^+^ and Cl^−^ in the serum were measured with an automatic biochemical analyser (Beckman Coulter, Brea, CA, USA). Liver samples collected from the control and PS-MPs treatment groups were fixed and embedded in paraffin. The embedded tissues were then sectioned, and the slices were stained with a hematoxylin-eosin (HE) staining kit (Sangon Biotech, Shanghai, China).

### 2.4. Cell Material and Growth Conditions

The human liver cells line HL7702 was obtained from Shanghai Chuanqiu Biotechnology (Shanghai, China), and the HepG2 cells line was kindly provided by the Shanghai Cancer Institute. Both of these cell lines were cultured in DMEM (Gibco, Carlsbad, CA, USA) supplemented with 10% foetal bovine serum (Gibco, Carlsbad, CA, USA) and 1% penicillin-streptomycin (Gibco, Carlsbad, CA, USA) in an incubator at 37 °C and 5% CO_2_. The cells were seeded in a cell culture flask, and the passage numbers were maintained at 5–30 for tests.

### 2.5. Cell Morphology Analysis

HL7702 and HepG2 cells were seeded in a 6-well microplate at a density of 1 × 10^5^ cells/well for 24 ± 0.5 h. Then, the cells were incubated with 0–2000 μg/cm^2^ PS-MPs or 300 μg/cm^2^ green, fluorescent labelled PS-MPs for 24 h. After incubation, all the cells were washed with PBS and observed with an EVOS™ imaging system (Invitrogen, Waltham, MA, USA).

### 2.6. Cytotoxicity Assay

The alamarBlue assay (Invitrogen, Waltham, MA, USA), which is used to analyse cell viability through the conversion of resazurin to red fluorescent resorufin via metabolically active cells, was used to confirm the cytotoxicity of the PS-MPs. The liver cells were seeded in a 96-well microplate at a density of 1 × 10^4^ cells/well and incubated for 24 ± 0.5 h. Then, 0–2000 μg/cm^2^ concentration gradient of PS-MPs was used to co-incubate with the cells for 24 h. After incubation, the cell culture supernatants were collected for lactate dehydrogenase (LDH) leakage, AST, and ALT assays. All the cells were washed with PBS, and 100 μL of fresh medium supplemented with 0.1 mg/mL alamarBlue was added to each well for 4 h in an incubator. The A570 and A600 were measured to calculate the cell viability.

For the LDH assay, the collected supernatants were centrifuged at 2000 r/min, and LDH leakage was measured with the LDH Cytotoxicity Detection Kit (Roche, Munich, Germany).

### 2.7. Enzyme-Linked Immunosorbent Assay

For the AST and ALT ELISA, the cell culture supernatants collected from the cytotoxicity assay were evaluated with the Human AST ELISA Kit and Human ALT ELISA Kit (Abcam, Cambridge, UK).

### 2.8. Flow Cytometry Bead-Based Assay

HL7702 and HepG2 cells were seeded in a 6-well micro plate and treated with 0, 100, 1000, or 2000 μg/cm^2^ PS-MPs for 24 h. After treatment, the cell culture supernatants were collected, and the cell lysates were lysed with lysis buffer (Millipore, Germany) after being rinsed by PBS. All the samples were centrifuged, and the concentrations of 12 cytokines (IL-1β, IL-2, IL-4, IL-5, IL-6, IL-8, IL-10, IL-12, IL-17, IFN-γ, IFN-α, and TNF-α) in the cell lysates and culture supernatants were evaluated with an ABplex Human Chemokine 12-Plex Assay Kit (ABclonal, Wuhan, China). The flow cytometry bead-based assays were performed in accordance with the manufacturer’s protocols.

### 2.9. Proteome Profiling Arrays

The Human Phosphorylation Pathway Profiling Array C55 (RayBio, Peachtree Corners, GA, USA) was used to detect the phosphorylation levels of proteins involved in the MAPK, AKT, JAK/STAT, NF-κB, and TGF-β signalling pathways. The prechilled lysis buffer was used to extract the total protein from PS-MPs-treated cells, and the BCA Assay Kit (Takara, Shiga, Japan) was used to determine the protein concentration. For each array, 200 µg of protein sample was incubated at 4 °C overnight. The bound proteins were detected via a Doc Molecular Imager (Bio-Rad, Hercules, CA, USA) according to the manufacturer’s instructions. The optical density value was determined by ImageJ and used to quantify the plots. The protein information is listed in the Supplementary Information (Tables S1–S6). The background, Negative Control spots (NEG), and Positive Control spots (POS) were used to normalize the results with the supplemented Excel sheet. The algorithm that can be used to calculate and determine the signal fold expression between similar analytes in Excel is ‘X(Ny) = X(y) × P1/P(y)’. P1 means signal density of Positive Control spots on the reference array; P(y) means signal density of Positive Control spots on array “y”; X(y) means signal density for spot “X” on array “y”; X(Ny) = normalized signal intensity for spot “X” on array “y”.

### 2.10. RNA Extraction and RT-qPCR Array

The total RNA was extracted from treated cells with TaKaRa MiniBEST Universal RNA Extraction Kit (TAKARA, Japan), and the NanoDrop2000 (Thermo Scientific, Waltham, MA, USA) was used to measure the RNA concentration. A total of 1 μg of total RNA was used to synthesize the cDNA at 65 °C for 5 min to dissolve the secondary structures and then anneal it with 5x RT Master Mix at 37 °C for 15 min, 50 °C for 5 min, and 98 °C for 5 min by using ReverTra Ace^®^ qPCR RT Master Mix (TOYOBO, Osaka, Japan). The template cDNA and Inflammatory Response & Autoimmunity PCR Array plate (WcGene Biotech, Shanghai, China) were used for quantitative reverse transcription PCR (RT-qPCR), which was conducted with a QuantStudio 7 Flex (Applied Biosystems, USA) and PowerUp™ SYBR™ Green Master Mix (Applied Biosystems, Waltham, MA, USA). All the qRT-PCR data were analysed by 2−ΔΔCt method, normalized by log, and compared with the control [16]. The heatmap was drawn with the HemI heatmap illustrator [17]. GAPDH was used as the reference, and information of the array plate is listed in Supplementary Information (Tables S1–S6).

### 2.11. Western Blot Analysis

The protease inhibitor cocktail (Beyotime, Shanghai, China) was added to prechilled RIPA lysis buffer (Millipore, Darmstadt, Germany) and used to extract total protein from PS-MPs-treated cells. All the extracted samples were centrifuged and then measured with a BCA Assay Kit. After being treated with LDS sample buffer (Invitrogen, Waltham, MA, USA) at 95 °C, all the samples were separated by SDS-PAGE and then transferred to a PVDF membrane (Bio-Rad, Hercules, CA, USA). After blocking, the membranes were incubated with diluted Akt, TAK1, TGF-β, p53, mTOR, P38 MAPK, Erk1/2, and GAPDH antibodies overnight at 4 °C. After incubation, the membranes were rinsed with TBS-T and then incubated with HRP-linked secondary antibodies for 1 h. An enhanced chemiluminescence (ECL) substrate (Thermo Scientific, Waltham, MA, USA) and Doc Molecular Imager were used to detect the bands. GAPDH was used as the reference protein, and the entire antibody was obtained from Cell Signaling Technology (CST, Danvers, MA, USA). The bands were determined by ImageJ (ver. 1.54b, NIH, Bethesda, MD, USA) to quantify the area under the curve (AUC).

### 2.12. Statistical Analysis

The benchmark dose (BMD) analyses were conducted using the Benchmark Dose Tools (BMDS) 3.3.2 [18], which was released by the U.S. Environmental Protection Agency (USEPA). BMD was calculated by using the Exponential or Hill continuous model as the curve model. The model that had the lowest AIC value was used to calculate the BMD values. All the BMDs were calculated at the 0.95 confidence level.

All of the data are expressed as the mean ± standard deviation (SD), and the results of three individual experiments were statistically analysed by one-way ANOVA followed by Dunnett’s test to determine the differences between groups. A *p* value ≤ 0.05 was considered to indicate a statistically significant difference.

## 3. Results

### 3.1. In Vivo Assay

#### Effects of PS-MPs on the Mouse

**Figure 1:**
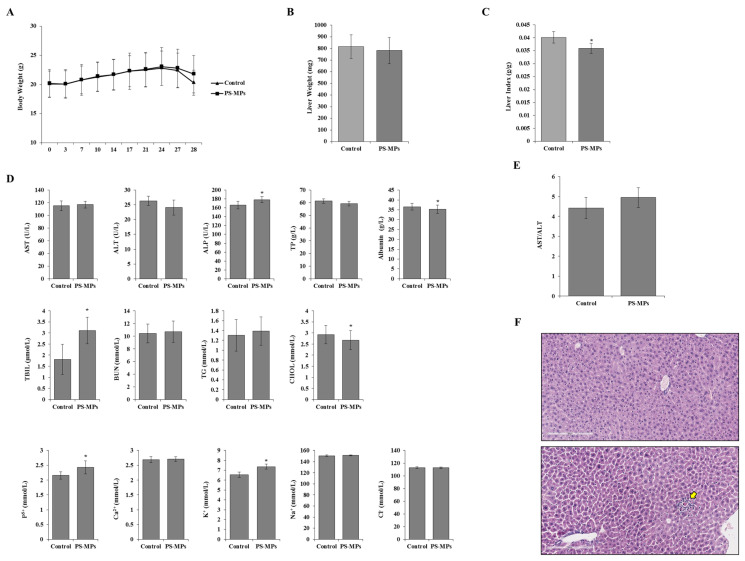
Effects of PS-MPs on mice. The (**A**) body weight; (**B**) liver weights; and (**C**) organ index after 28 days of PS-MPs exposure; (**D**) blood biochemical examination of the serum; (**E**) ratio of AST/ALT; (**F**) H&E staining of the liver tissue. Yellow arrow: perivascular infiltration of liver tissue; *: *p* ≤ 0.05; scale bar: 200 μm.

The body and liver weights of the mice after PS-MPs exposure are shown in Figure 1. After oral exposure to 0.5 mg/day of 0.1 μm PS-MPs, there was no significant difference in body weight between the control and PS-MPs-treated groups after 28 days (Figure 1A). Although the liver weights did not differ between the control and PS-MPs treatment groups, the liver indices (organ/body weight) were significantly lower in the PS-MPstreated groups than in the control group (Figure 1C). For the blood biochemical examination of the serum, the ALP, TBIL, P^5+^, and K^+^ levels also increased after 28 d of exposure to PS-MPs (Figure 1D). Compared with that of the control group, the liver tissue of the experimental group was similar except for weak perivascular infiltration, which may have occurred occasionally (Figure 1F).

### 3.2. In Vitro Assay

#### 3.2.1. Effects of PS-MPs on Cell Morphology

**Figure 2:**
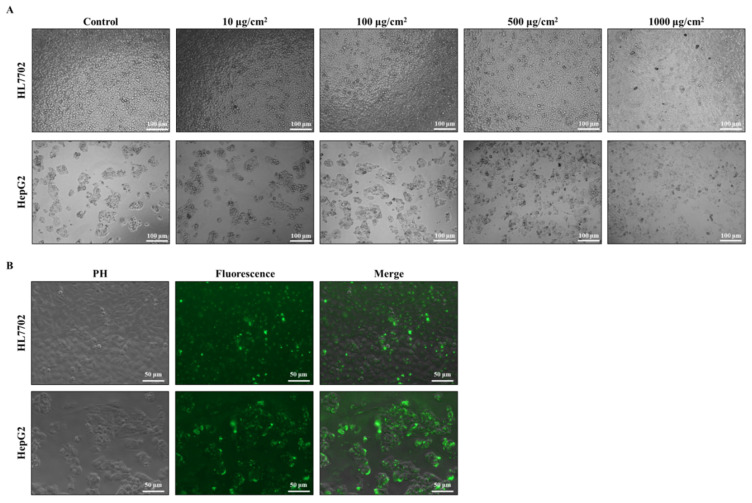
PS-MPs enter and cause changes in liver cell morphology. (**A**) Cell morphology after treatment with different PS-MP doses. Scale bar: 100 μm. (**B**) The location of green, fluorescent PS-MPs in liver cells. Scale bar: 50 μm.

As the results show in Figure 2, the HL7702 cells presented a disordered arrangement, while both surfaces of the two cell lines were rough, and the boundaries were unclear, which was accompanied by an increased dose of PS-MPs (Figure 2A). To clarify whether 0.1 μm PS-MPs enter liver cells or not, 300 μg/cm^2^ green, fluorescent PS-MPs were used to treat the cells. The results show that the green fluorescence was located within the cells after the cells were washed with PBS (Figure 2B).

#### 3.2.2. Cytotoxic Effects

**Figure 3:**
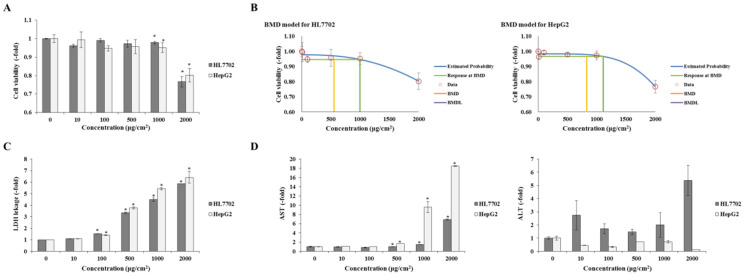
Cell viability of PS-MPs in liver cells. (**A**) Cell viability of two liver cell lines after PS-MP treatment; (**B**) BMD model of cell viability for HL7702 and HepG2 cells; (**C**) LDH leakage for HL7702 and HepG2 cells; (**D**) AST and ALT levels in HL7702 and HepG2 cells. *: *p* ≤ 0.05; blue line: estimated probability line between cell viability and treatment dose; light green line: the response value of cell viability at BMD; red circle: The cell viability and treatment dose data; green line: the BMD value; yellow line: the BMDL value.

The results of the alamarBlue cell viability assay are shown in Figure 3. Compared with that of the control, the cell viability of the HL7702 and HepG2 cells decreased in a dose-dependent manner after PS-MPs treatment. Both of these two liver cells exhibited a significant decrease when exposed to PS-MPs at 1000 μg/cm^2^, whereas the cell viability decreased by approximately 20% at 2000 μg/cm^2^ (Figure 3A). In addition, benchmark dose (BMD) modelling, which is used to determine the point of departure for risk assessment and is recommended by the USEPA and EFSA, showed that the effective points (0.95 confidence level) of PS-MPs in HL7702 and HepG2 cells were 1109.58 and 998.26 μg/cm^2^, similar to the results of cell viability (Figure 3B). The LDH leakage also corresponded to the results of cell viability, and the LDH leakage in both cell lines significantly increased at 100 μg/cm^2^ (Figure 3C). In addition, the AST content in the cell culture supernatant also showed a dose-dependent increase, while the ALT had no significant difference from the control (Figure 3D).

#### 3.2.3. Effect of PS-MPs on Chemokines

**Figure 4:**
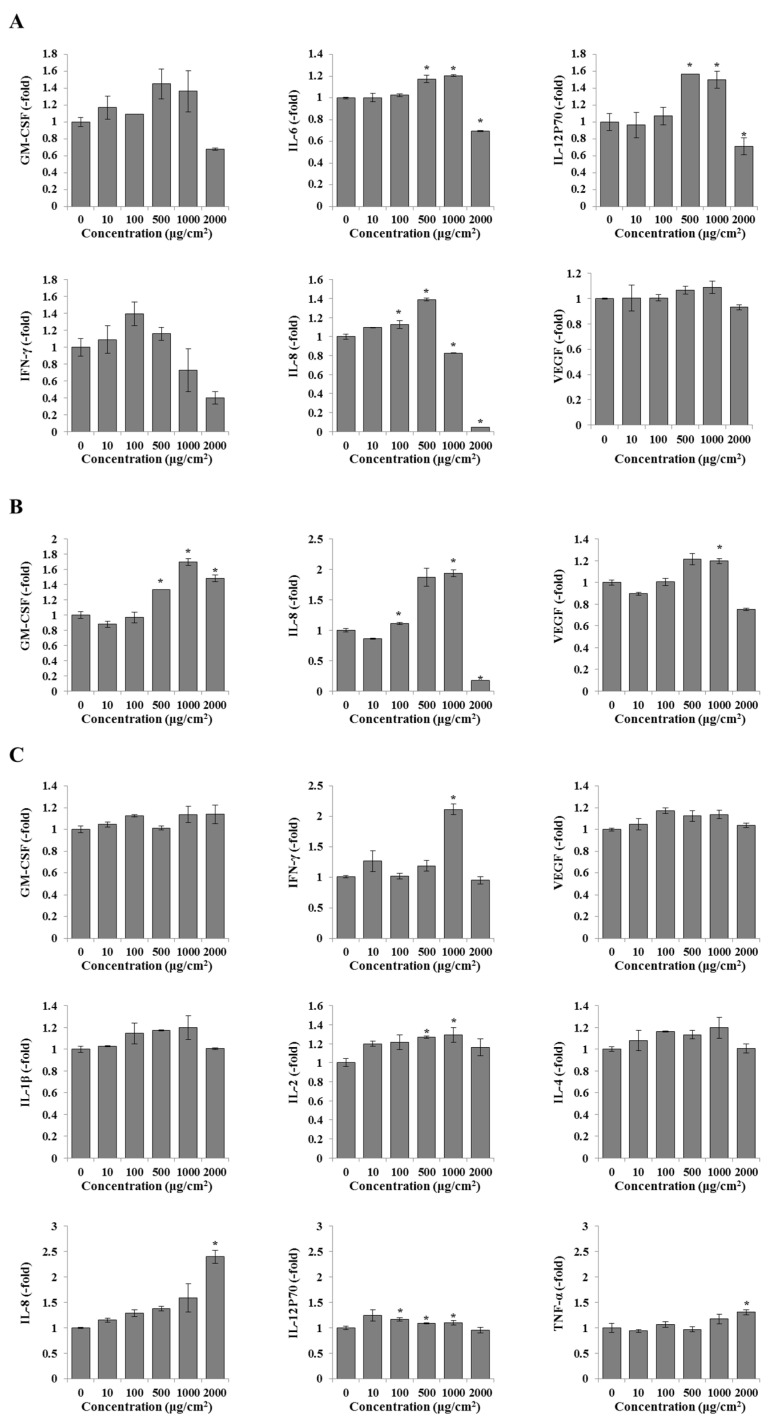
Effects of PS-MPs on the levels of chemokines in liver cells. (**A**) Relative levels in the cell culture medium of HL7702 cells. (**B**) The relative levels in the cell culture medium of HepG2 cells. (**C**) The relative levels in the lysates of HepG2 cells. *: *p* ≤ 0.05.

The results of relative chemokine levels are shown in Figure 4. For the cell culture supernatant, the relative levels of GM-CSF, IFN-γ, IL-6, IL-8, and IL-12P70 were increased in HL7702 cells (Figure 4A), whereas only the levels of GM-CSF, IL-8, and VEFG were increased in HepG2 cells (Figure 4B) after exposure to PS-MPs. For cell lysates (Figure 4C), the levels of IFN-γ, IL-1β, IL-2, IL-4, IL-8, IL-12P70, and TNF-α were increased in HepG2 cells after exposure to PS-MPs.

**Figure 5:**
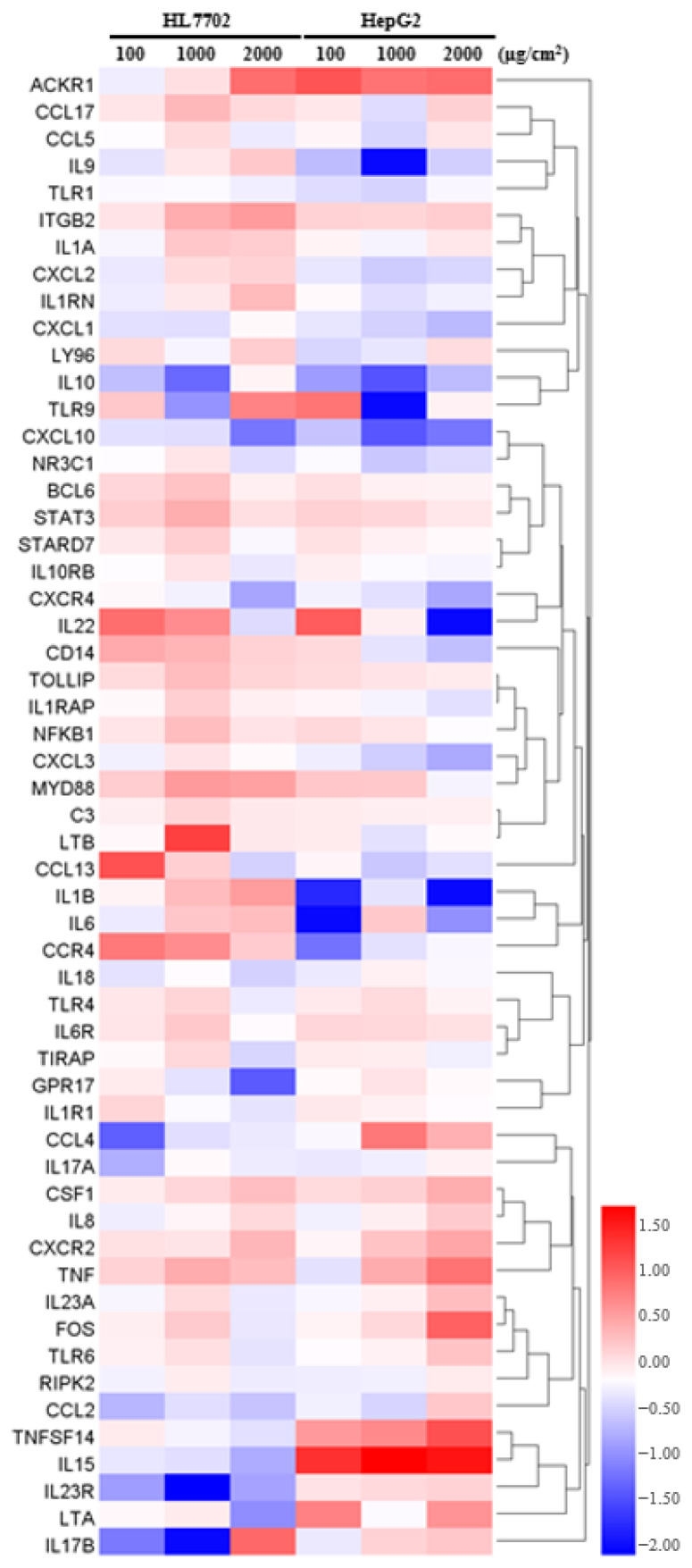
Heatmap of the inflammatory response and autoimmunity gene expression levels. The heatmap shows the relative expression levels of the genes. The red colour indicates upregulated genes, and the blue colour indicates downregulated genes.

We also used a qPCR array to confirm the relative expression levels of chemokine genes after PS-MPs exposure (Figure 5). Compared with those of the controls, the relative *ACKR1, CCL17, ITGB2, STAT3, CSF1, TNF, IL17B, TOLLIP, IL6R, MYD88, NFKB1,* and *CXCR2* levels were upregulated, whereas the *CXCL10, CXCR4, NR3C1, IL18, RIPK2, IL10, IL1R1, TLR1,* and *CXCL1* levels were downregulated after PS-MPs exposure.

#### 3.2.4. Detection of the Protein and Phosphorylation Levels of the Inflammation-Related Signalling Pathway

**Figure 6:**
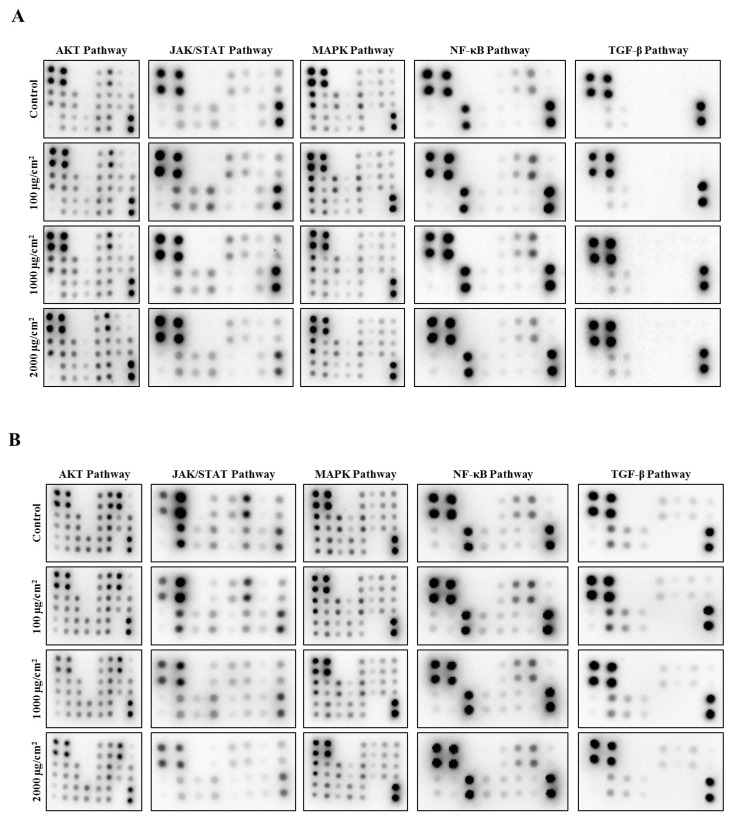

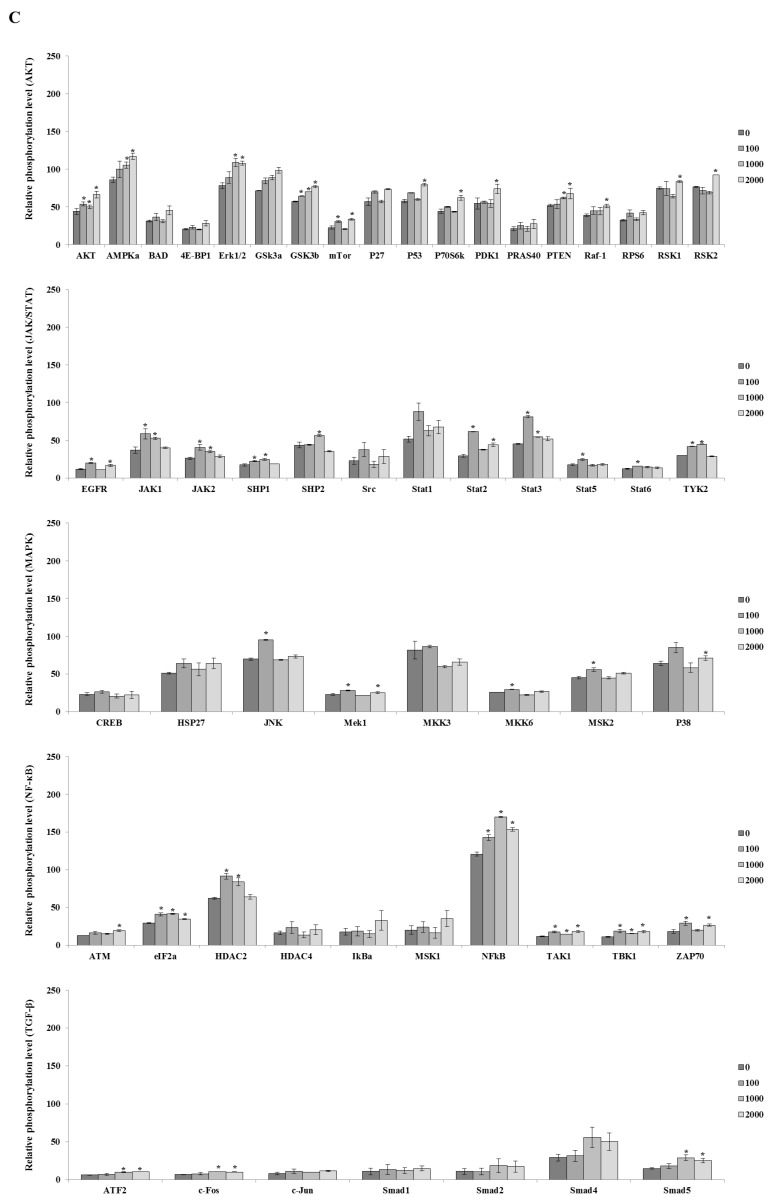

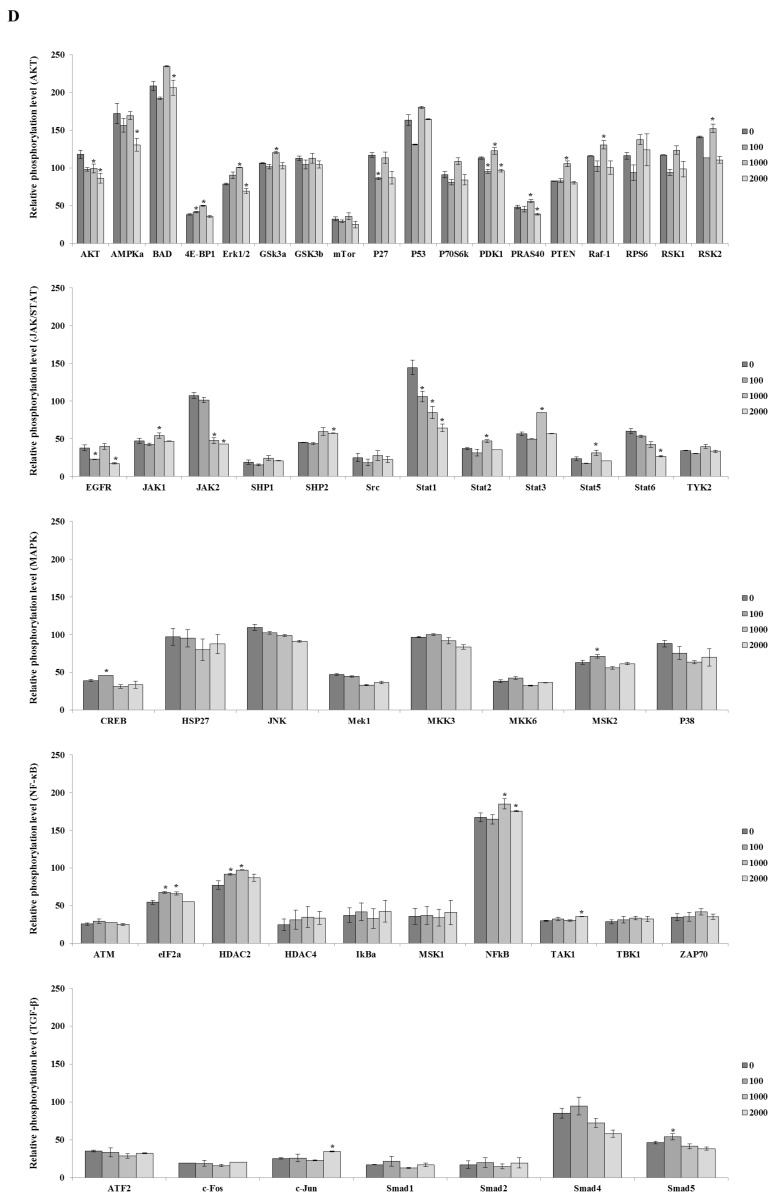
Effects of PS-MPs on the phosphorylation levels of proteins in 5 signalling pathways. The results of the antibody microarray (**A**) and relative phosphorylation levels (**C**) in HL7702 cells. The results of the antibody microarray (**B**) and relative phosphorylation levels (**D**) in HepG2 cells. *: *p* ≤ 0.05.

After exposure to PS-MPs, total proteins from HL7702 and HepG2 cells were extracted with cold RIPA buffer, and the phosphorylation levels of proteins in the AKT, JAK-STAT, MAPK, NF-κB, and TGF-β signalling pathways were detected by using an antibody microarray. Compared with those of the controls, the phosphorylation levels of AKT, AMPKa, Erk1/2, GSK3b, mTor, P53, P70S6k, PDK1, PTEN, Raf-1, RSK1, and RSK2 in the AKT signalling pathway; JAK1, JAK2, Stat2, Stat3, and TYK2 in the JAK-STAT signalling pathway; eIF2a, HDAC2, NF-κB, TAK1, TBK1, and ZAP70 in the NF-κB signalling pathway; and ATF2 and c-Fos in the TGF-β signalling pathway were increased in HL7702 cells (Figure 6A). The phosphorylation levels of 4E-BP1, Erk1/2, GSK3a, PRAS40, PTEN, Raf-1, and RSK2 in the AKT signalling pathway; JAK1, SHP2, Stat2, Stat3, and Stat5 in the JAK-STAT signalling pathway; eIF2a, HDAC2, NF-κB, and TAK1 in the NF-κB signalling pathway; and c-Jun in the TGF-β signalling pathway were increased in HepG2 cells (Figure 6B).

**Figure 7:**
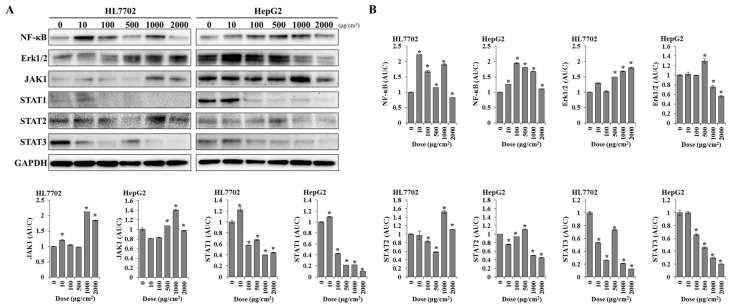
The protein expression levels of NF-κB, Erk1/2, JAK1, STAT1, STAT2, STAT3, and GAPDH after exposure to PS-MPs. (**A**) Western blot at 24 h after exposure to PS-MPs and (**B**) the area under the curve of protein expression. *: *p* ≤ 0.05.

Furthermore, the results of the Western blot assay are shown in Figure 7. For HL7702 cells, the expression levels of NF-κB p65 were increased after exposure to low PS-MPs concentrations and decreased with increasing concentrations. The expression levels of Erk1/2 showed a dose-dependent upregulation. Both JAK1 and STAT2 were upregulated after 1000 μg/cm^2^ PS-MPs exposure, while STAT1 and STAT3 showed a dose-dependent downregulation. For HepG2 cells, the expression patterns of NF-κB p65, JAK1, STAT1, STAT2, and STAT3 were similar with those of HL7702, while those of Erk1/2 were increased at 500 μg/cm^2^ but decreased at 1000 μg/cm^2^, compare with the control.

**Figure 8:**
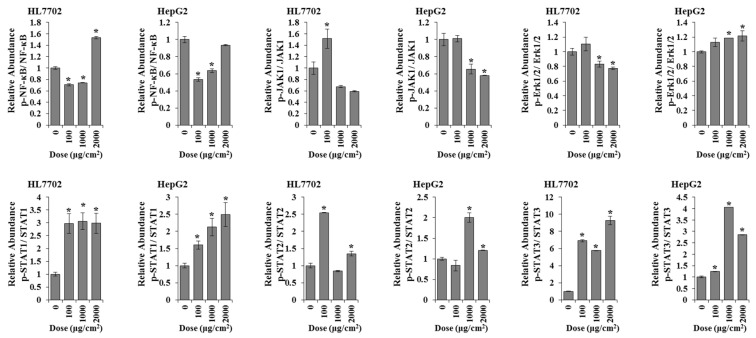
The ratios of p-NF-κB, NF-κB, p-Erk1/2, Erk1/2, p-JAK1, JAK1, p-STAT1, STAT1, p-STAT2, STAT2, p-STAT3, and STAT3. The protein expression was quantified via densitometry and normalized to that of GAPDH. *: *p* ≤ 0.05.

We also found that the expression levels of p-Erk1/2/Erk1/2 in HepG2 cells and p-STAT1/STAT1, p-STAT2/STAT2, and p-STAT3/STAT3 in both of the two cells were significantly increased after PS-MPs exposure (Figure 8).

## 4. Discussion

With improvements in people’s quality of life, the global plastic production and consumption continue to increase. In 2020, the global production of plastics was approximately 367 million tons, and the output is predicted to reach 1.1 billion tons by 2050. The Norwegian Environment Agency reported that MPs pollution mainly originates from a wide range of sources, including tire wear, synthetic textiles, plastic production industry pollution, urban dust, ship coatings, and personal care products. Although MPs are considered to cause pollution hazards to various waters such as oceans, rivers, or lakes, recent studies have shown that various environments, including the atmosphere and soil, are also polluted by different degrees of MPs [19]. A series of studies have shown that exposure to MPs can aggravate the dysregulated expression of oxidative stress-related genes and activate the Nrf-2 signalling pathway in marine vertebrates and invertebrates [20]. Ingestion of MPs by the human body may also cause oxidative stress, inflammatory responses, and immune responses.

In this study, PS-MPs with a particle size of 0.1 μm were used to investigate the effects on liver tissue and function in mice. The effects on cell viability, LDH leakage, ALT, AST, expression levels of chemokine, and phosphorylation levels of the inflammatory signalling pathway were also confirmed in the HL7702 and HepG2 human liver cell lines after exposure to PS-MPs. The results showed that PS-MPs decreased the ratios of liver weight to body weight by 10.7%. The ALP, TBIL, phosphorus, and potassium levels also increased, whereas the serum albumin and CHOL levels decreased. Although the differences in AST and ALT in the serum of the control and PS-MPs-treated groups were not significant, the AST/ALT ratio tended to increase after PS-MPs treatment. AST, ALT, albumin, and TBIL are important indices of liver function and are closely related to liver diseases, such as hepatic fibrosis [21].

To clarify the effects of 0.1 μm PS-MPs on the liver, the HL7702 and HepG2 cell lines were used to confirm the adverse effects. After exposure to 100 μg/cm^2^ PS-MPs, two liver cell lines started to show rough and unclear boundaries, and the disordered arrangement of HL7702 cells and the loss of ‘island’ growth characteristics of HepG2 cells gradually increased with increasing exposure doses. On the basis of these results, we used 0.1 μm green fluorescently labelled PS-MPs to confirm whether the PS-MPs could enter the cells. As shown in Figure 3B, 0.1 μm PS-MPs can enter HL7702 and HepG2 cells and cause direct cytotoxicity. Thus, the cytotoxicity was subsequently confirmed. Both the HL7702 and HepG2 cells showed a dose-dependent decrease in cell viability after exposure to 0.1 μm PS-MPs. The results also showed that the 1000 μg/cm^2^ PS-MPs caused a significant decrease in the levels of two liver cells and resulted in approximately 20% cell death under the 2000 μg/cm^2^ exposure condition. We also used a BMD model recommended by the USEPA to calculate the dose of adverse effects, and the results revealed that exposure to 1109.58 and 998.26 μg/cm^2^ PS-MPs may cause adverse effects on HL7702 and HepG2 cells, respectively. This value was also similar to our experimental result. Correspondingly, the LDH leakage and AST in the cell culture supernatant significantly increased in a dose-dependent manner, which verified the cytotoxicity of 0.1 μm PS-MPs. The LDH leakage increased 1.53–5.86- and 1.40–6.42-fold in the HL7702 and HepG2 cells, and the AST values in the serum were 1.45–6.88- and 1.69–18.49-fold in the HL7702 and HepG2 cells, respectively. Based on these results, although the results in mice revealed weak injury to the liver, 0.1 μm PS-MPs can enter liver cells, cause cytotoxicity, and lead to liver function damage.

We also confirmed the protein and mRNA expression levels of chemokines in both of the two liver cell lines after PS-MPs exposure. The expression levels of cytokines such as IL-6, IL-8, and IL-12P70 were increased in the HL7702 cells, and GM-CSF, VEGF, and IL-8 were increased in the HepG2 cell culture supernatant, whereas the levels of IL-2, IL-8, IL-12P70, TNF-α, and IFN-γ were increased in the HepG2 cell lysate. We also noted that almost all of these cytokine levels increased in a dose-dependent manner after PS-MPs exposure but decreased at 2000 μg/cm^2^, and the expression level of IL-8 was significantly increased in the culture supernatants of both cell lines after exposure to PS-MPs. As a member of the platelet factor 4 cytokine family, IL-8 can be induced by inflammatory stimuli and synthesized at these sites to attract and activate neutrophils [22,23]. The increased IL-6 and TNF-α levels in the PS-MPs treatment groups suggested that exposure to PS-MPs may induce an inflammatory response in HL7702 and HepG2 cells. IL-6 is also rapidly induced by infections and injuries and can promote host defence by stimulating the acute phase, hematopoietic, and immune responses. Dysregulation of IL-6 can also cause chronic inflammation and autoimmune diseases [24].

In addition to these cytokines, the results of the qPCR array also showed the expression levels of *IL-6R, CCL17,* and *ACKR1*, which are related to inflammatory responses or autoimmune diseases [25,26]. The *ITGB2, CSF1,* and *CXCR2* genes are closely related to cancer. The expression levels of *IL17B, TLR4,* and *MYD88*, which are related to the *NF-κB* pathway, were also increased by PS-MPs, and the *NFKB1* expression level was also increased. [27,28]. The *IL17B* increased 9.26- and 1.75-fold after the addition of 2000 μm/cm^2^ PS-MPs to HL7702 and HepG2 cells, respectively. *TLR4* also increased 1.36- and 1.22-fold, and *MYD88* increased 3.84- and 1.67-fold after 1000 μm/cm^2^ PS-MPs exposure, respectively. The *NFKB1* expression level was increased 2.09-fold in HL7702 cells and 1.29-fold in HepG2 cells exposed to 1000 and 100 μm/cm^2^ PS-MPs, respectively. Although the results of the qPCR array suggested that the NF-κB pathway may be activated, we also used a Western blot analysis to confirm the protein expression levels. Similar to the results of mRNA expression, the NF-κB expression increased 2.22- and 1.95-fold with 10 and 100 μm/cm^2^ PS-MPs exposure, respectively, and then decreased in a dose-dependent manner to a similar level as that of the control.

After confirming the phosphorylation levels of key factors in the AKT, JAK-STAT, MAPK, NF-κB, and TGF-β signalling pathways, the phosphorylation levels of JAK1, Stat2, and Stat3 (JAK-STAT signalling pathway) as well as eIF2a, HDAC2, NF-κB, and TAK1 (NF-κB signalling pathway) were increased in both HL7702 and HepG2 cells (Figure 6B). The relative abundances of p-NF-κB/NF-κB and p-JAK1/JAK1 in HL7702 cells and p-STAT1/STAT1, p-STAT2/STAT2, and p-STAT3/STAT3 in both of the two cell lines were also increased after PS-MPs exposure.

As one of the most important triggers of the NF-κB pathway, TLR4 can be activated by multiple exogenous and endogenous factors and then bind to MyD88 to activate NF-κB. Thus, NF-κB can be transferred to the nucleus and then increase the levels of pro-inflammatory cytokines such as TNF-α, IFN-γ, IL-1, IL-5, IL-6, IL-8, and IL-17 [29,30,31,32]. In this study, the expressions of IL-6, IL-8, and TNF-α were induced by PS-MPs via the NF-κB pathway, which subsequently activated the JAK/STAT signalling pathway. The JAK/STAT signalling pathway is a key pathway involved in cell development, proliferation, metabolism, infection, inflammation, and immune response regulation [33]. Usually, when cells respond to external stimuli such as inflammation or oxidative stress, cytokines are released, bind to receptors, and then transmit signals to promote the activation of JAK receptors and tyrosine phosphorylation. Phosphorylated JAK then activates and promotes the transition of STATs to pSTATs. Subsequently, pSTATs form dimers and translocate to the nucleus, where they bind to target DNA and regulate the expression of genes related to inflammation, proliferation, and metabolism [34].

## 5. Study Limitations

In this study, two crucial limitations should be noted. One limitation is the use of PS-MPs microspheres. Although this this material has been wildly used in related studies, it is not representative of the most prevalent plastics found under realistic environmental conditions. Another limitation is that the PS-MPs microspheres have a single size and regular circle, whereas the nano- and microplastics in actual external environments have diverse sizes, shapes, polymers and even wrappings in various chemicals.

## 6. Conclusions

In conclusion, our results revealed four major findings related to 0.1 μm PS-MPs and their effects on the liver: (1) exposure to 0.1 μm PS-MPs can decrease the liver index and increase AST/ALT values in mice; (2) 0.1 μm PS-MPs can enter cells and induce cytotoxicity with decreased cell viability and increased AST; (3) inflammation was induced by 0.1 μm PS-MPs, and cytokines such as GM-CSF, IL-6, IL-8, and IL-12p70 were significantly increased; and (4) the expression and phosphorylation levels of factors in the NF-κB and JAK1 pathways were increased, which suggest that the TLR4/MyD88/NF-κB and JAK-STAT pathways were activated in liver cells. Overall, the results of this study can reiterate the potential risk of the new environmental pollutant, PS-MPs, and provide important information concerning inflammation and potential health hazards to the liver.

## Data Availability

The original contributions presented in the study are included in the article, further inquiries can be directed to the corresponding authors.

## Supplementary Materials

The following supporting information can be downloaded at: https://www.mdpi.com/article/10.3390/toxics12120932/s1, Table S1: The site information of MAPK pathway phosphorylation array; Table S2: The site information of AKT pathway phosphorylation array; Table S3: The site information of JAK-STAT pathway phosphorylation array; Table S4: The site information of NF-κB pathway phosphorylation array; Table S5: The site information of TGF-β pathway phosphorylation array; Table S6: The site information of Inflammatory Response & Autoimmunity PCR Array.
