# Supplementary material for: PS-MPs Induced Inflammation and Phosphorylation of Inflammatory Signalling Pathways in Liver
Source: Toxics. 2024 Dec 22;12(12):932. doi: 10.3390/toxics12120932 (PMC11679018; doi:10.3390/toxics12120932)
Supplement: Supplementary file 1 [file toxics-12-00932-s001.zip › toxics-3355756-supplementary.pdf]

The site information of proteome profiling arrays

Table S1. The site information of MAPK pathway phosphorylation array

MAPK Pathway	A	B	C	D	E	F	G	H
1	POS	POS	Neg	Neg	AKT	CREB	Erk1/2	GSK3a
2	POS	POS	Neg	Neg	AKT	CREB	Erk1/2	GSK3a
3	GSK3b	HSP27	JNK	Mek1	MKK3	MKK6	MSK2	mTor
4	GSK3b	HSP27	JNK	Mek1	MKK3	MKK6	MSK2	mTor
5	P38	P53	P70S6k	RSK1	RSK2	Neg	Neg	POS
6	P38	P53	P70S6k	RSK1	RSK2	Neg	Neg	POS

Table S2. The site information of AKT pathway phosphorylation array

AKT Pathway	A	B	C	D	E	F	G	H
1	POS	POS	Neg	Neg	AKT	AMPKa	BAD	4E-BP1
2	POS	POS	Neg	Neg	AKT	AMPKa	BAD	4E-BP1
3	Erk1/2	GSk3a	GSK3b	mTor	P27	P53	P70S6k	PDK1
4	Erk1/2	GSk3a	GSK3b	mTor	P27	P53	P70S6k	PDK1
5	PRAS40	PTEN	Raf-1	RPS6	RSK1	RSK2	Neg	POS
6	PRAS40	PTEN	Raf-1	RPS6	RSK1	RSK2	Neg	POS

Table S3. The site information of JAK-STAT pathway phosphorylation array

JAK/STAT Pathway	A	B	C	D	E	F	G	H
1	POS	POS	Neg	EGFR (Ser1070)	JAK1 (Tyr1022)	JAK2 (Tyr1007/1008)	SHP1 (Ser591)	SHP2 (Tyr542)
2	POS	POS	Neg	EGFR (Ser1070)	JAK1 (Tyr1022)	JAK2 (Tyr1007/1008)	SHP1 (Ser591)	SHP2 (Tyr542)
3	Src(Tyr419)	Stat1 (Ser727)	Stat2 (Tyr689)	Stat3 (Tyr705)	Stat5 (Tyr694)	Stat6 (Tyr641)	TYK2 (Tyr1054)	Pos
4	Src(Tyr419)	Stat1 (Ser727)	Stat2 (Tyr689)	Stat3 (Tyr705)	Stat5 (Tyr694)	Stat6 (Tyr641)	TYK2 (Tyr1054)	Pos

Table S4. The site information of NF-κB pathway phosphorylation array

NFkB Pathway	A	B	C	D	E	F	G	H
1	POS	POS	NEG	NEG	ATM (S1981)	eIF2a (S51)	HDAC2 (S394)	HDAC4 (S632)
2	POS	POS	NEG	NEG	ATM (S1981)	eIF2a (S51)	HDAC2 (S394)	HDAC4 (S632)
3	IkBa (S32)	MSK1 (S376)	NFkB (S536)	Stat1 (S727)	TAK1 (S412)	TBK1 (S172)	ZAP70 (Y292)	POS
4	IkBa (S32)	MSK1 (S376)	NFkB (S536)	Stat1 (S727)	TAK1 (S412)	TBK1 (S172)	ZAP70 (Y292)	POS

Table S5. The site information of TGF-β pathway phosphorylation array

TGFb Pathway	A	B	C	D	E	F	G	H
1	Pos	Pos	Neg	Neg	ATF2	c-Fos	c-Jun	Smad1
2	Pos	Pos	Neg	Neg	ATF2	c-Fos	c-Jun	Smad1
3	Smad2	smad4	Smad5	TAK1	Neg	Neg	Neg	Pos
4	Smad2	smad4	Smad5	TAK1	Neg	Neg	Neg	Pos

The information of RT-qPCR array

Table S6. The site information of Inflammatory Response & Autoimmunity PCR Array

PCR Array	1	2	3	4	5	6	7	8	9	10	11	12
**A**	ACKR1	CCL17	CCL4	CCR7	CXCL10	CXCR4	IL17A	IL22	ITGB2	NR3C1	TLR2	TNFSF14
**B**	ACKR2	CCL19	CCL5	CD14	CXCL2	FASLG	IL17B	IL23A	KNG1	PTGS2	TLR3	TOLLIP
**C**	BCL6	CCL2	CCL7	CD40	CXCL3	FOS	IL18	IL23R	LTA	RIPK2	TLR4	IL1RN
**D**	C3	CCL21	CCL8	CD40LG	CXCL5	GPR17	IL1A	IL5	LTB	SELE	TLR5	IL9
**E**	C3AR1	CCL22	CCR1	CEBPB	CXCL6	IFNG	IL1B	IL6	LY96	STARD7	TLR6	NOS2
**F**	CCL11	CCL23	CCR2	CRP	CXCL9	IL10	IL1R1	IL6R	MYD88	STAT3	TLR7	TLR1
**G**	CCL13	CCL24	CCR3	CSF1	CXCR1	IL10RB	IL1RAP	IL8	NFKB1	TIRAP	TLR9	TNF
**H**	CCL16	CCL3	CCR4	CXCL1	CXCR2	IL15	ACTB	GAPDH	HPRT1	18S	NTC	NTC
